# Elevated blood pressure in youth in pediatric weight management programs in the Pediatric Obesity Weight Evaluation Registry (POWER)

**DOI:** 10.1111/jch.14423

**Published:** 2022-01-31

**Authors:** Helen J. Binns, Madeline Joseph, Adolfo J. Ariza, Suzanne E. Cuda, Asheley C. Skinner, Haolin Xu, Jared M. Tucker, Sarah E. Hampl, Melissa Santos, Shawyntee Mayo, Eileen C. King, Shelley Kirk, the POWER Work Group

**Affiliations:** ^1^ Pediatrics Ann & Robert H. Lurie Children's Hospital of Chicago Northwestern University Feinberg School of Medicine Chicago Illinois USA; ^2^ College of Medicine University of Florida Jacksonville Florida USA; ^3^ Children's Hospital of San Antonio Baylor College of Medicine San Antonio Texas USA; ^4^ Population Health Sciences Duke University School of Medicine Durham North Carolina USA; ^5^ Duke Clinical Research Institute Durham North Carolina USA; ^6^ Helen DeVos Children's Hospital Michigan State University College of Human Medicine Grand Rapids Michigan USA; ^7^ Children's Mercy Hospital University of Missouri‐Kansas City School of Medicine Kansas City Missouri USA; ^8^ Connecticut Children's Hartford Connecticut USA; ^9^ Pediatrics Division of Biostatistics and Epidemiology Cincinnati Children's Hospital Medical Center Cincinnati Ohio USA; ^10^ Pediatrics College of Medicine University of Cincinnati Cincinnati Ohio USA; ^11^ The Heart Institute Center for Better Health and Nutrition Cincinnati Children's Hospital Medical Center Cincinnati Ohio USA

**Keywords:** blood pressure, hypertension, obesity, pediatric weight management, pediatrics

## Abstract

Blood pressure (BP) assessment and management are important aspects of care for youth with obesity. This study evaluates data of youth with obesity seeking care at 35 pediatric weight management (PWM) programs enrolled in the Pediatric Obesity Weight Evaluation Registry (POWER). Data obtained at a first clinical visit for youth aged 3–17 years were evaluated to: (1) assess prevalence of BP above the normal range (*high BP*); and (2) identify characteristics associated with having *high BP* status. Weight status was evaluated using percentage of the 95^th^ percentile for body mass index (%BMIp95); %BMIp95 was used to group youth by obesity class (class 1, 100% to < 120% %BMIp95; class 2, 120% to < 140% %BMIp95; class 3, ≥140% %BMIp95; class 2 and class 3 are considered severe obesity). Logistic regression evaluated associations with *high BP*. Data of 7943 patients were analyzed. Patients were: mean 11.7 (SD 3.3) years; 54% female; 19% Black non‐Hispanic, 32% Hispanic, 39% White non‐Hispanic; mean %BMIp95 137% (SD 25). Overall, 48.9% had *high BP* at the baseline visit, including 60.0% of youth with class 3 obesity, 45.9% with class 2 obesity, and 37.7% with class 1 obesity. Having *high BP* was positively associated with severe obesity, older age (15–17 years), and being male. Nearly half of treatment‐seeking youth with obesity presented for PWM care with *high BP* making assessment and management of BP a key area of focus for PWM programs.

## 1 INTRODUCTION

Child/adolescent obesity strongly tracks into adulthood^1^, ^2^ and conveys an increased risk for morbidity and mortality of cardiovascular disease in adulthood, with the strength of the relationship to risk for cardiovascular disease increasing as the child ages.^3^, ^4^ Obesity is an established risk factor for hypertension in youth and risk increases with severity of obesity.^5^ A systematic review of 19 studies reported that youth with elevated blood pressure (BP) were more likely to have increased risk for markers of cardiovascular disease (such as high pulse wave velocity, high carotid intima‐media thickness, left ventricular hypertrophy) in adulthood.^6^ Thus, recognition of elevated BP by standardized BP measurement, and interpretation and early management are important aspects of optimal clinical care for youth with obesity.

Using current guideline interpretations for BP,^7^ national samples report 27.5% of youth ages 12–19 years with obesity had BP above a normal range (ie, elevated, stage 1, or stage 2 classification; henceforth, *high BP*); and 30.6% of youth with severe obesity (defined as body mass index [BMI]‐for‐age ≥120% of the 95^th^ percentile) had *high* *BP*.^8^ A recent report evaluated BP among national samples of youth ages 8–12 years and 13–17 years, reporting 2–3 times higher risk for *high BP* among youth with obesity as compared to youth of normal weight, but youth with severe obesity were not separately evaluated.^9^

Because treatment of *high* BP in childhood could potentially reduce long‐term cardiovascular risks, it is of particular importance to understand prevalence and risks for *high BP* among youth with obesity in the clinical setting to plan for their evaluation and management needs. This study evaluates data obtained in the Pediatric Obesity Weight Evaluation Registry (POWER). POWER is a consortium of multicomponent pediatric weight management (PWM) programs across the United States which prospectively enrolls patients seeking obesity management care and tracks parameters of care in an aggregate database.^10^ Three‐quarters of youth in POWER present with severe obesity.^11^ This study aims to evaluate data from youth enrolled in POWER to: (1) assess prevalence of *high BP* in the clinical setting among care‐seeking youth with obesity; and (2) identify characteristics associated with *high BP* status at a baseline visit.

## 2 METHODS

POWER was established in 2014 with the aim to better understand the complex nature of PWM and to collectively evaluate and improve medical care strategies for children and adolescents with obesity and improve health outcomes.^10^ The Data Coordinating Center for POWER is located at Cincinnati Children's Hospital Medical Center. POWER leadership supports POWER's infrastructure, which include an aggregate database and collaborative work, such as research and educational webinars. Financial support for POWER is provided via enrollment fees paid biannually by individual sites, with oversight provided by POWER's administrative staff at Cincinnati Children's Hospital Medical Center.

The Cincinnati Children's Hospital Institutional Review Board approved this study. Additionally, Institutional Review Boards at each site approved the study and monitored the site‐specific consent/assent process. The POWER study is registered with Clinical.Trials.gov (NCT02121132).

Patients evaluated were prospectively recruited from 35 participating sites in May 2014 through December 2017. Not all sites participated for the entire data period, as some joined POWER later during the study period.

Each site contributed data to a central database. Database elements included patient demographics (race, ethnicity, insurance type) and characteristics (sex, age, weight, height, BP). One BP measurement was entered for each patient visit; documentation of the BP measurement procedure (eg, electronic or manual measure, or if it was a report of an average of repeated BP measurements) was not obtained. Additionally, there was no information on BP measurement practices as protocols by site were not available; however, POWER did provide written BP assessment recommendations to sites based on Centers for Disease Control and Prevention (CDC) guidelines^12^ within a Data Definitions Document. The registry included optional data entry for patient diagnoses/conditions (eg, hypertension) existing at the time of the initial PWM visit and for medications/treatments (eg, antihypertensive medications) at the time of the initial PWM visit. However, these fields were not uniformly completed so were not considered in analyses. Thus, some youth included in these analyses had hypertension and may been taking antihypertensive medication before their initial PWM visit.

Data of patients aged 3–17 years with valid BP, height and weight measures, and body mass index (BMI) for age ≥95^th^ percentile at an initial visit were evaluated. Because BP guidelines lack interpretations for patients at extreme height‐for‐age (height z‐score [HtZ] > 3.9 or < ‐ 3.9), patients with height‐for‐age extreme values at their initial visit were excluded from these analyses.

Among 8933 active POWER participants, 990 (11.1%) were excluded from analyses; initial visit data of 7943 patients (88.9%) were analyzed. There were 107 (1.2%) excluded due to age missing, age < 3 years, or age ≥18 years, 509 (5.7%) excluded due to missing height or weight or BP, 299 (3.3%) had BMI < 95^th^ BMI percentile, and 75 (0.8%) had extreme height‐for‐age values.

Data were used to compute percentage of the 95^th^ percentile for BMI (%BMIp95) and height z‐score (HtZ) based on CDC 2000 references.^13^ BP interpretations were done using references in the 2017 guideline (SAS macro).^7^ Obesity class groups were defined as follows: class 1, 100% to < 120% %BMIp95; class 2, 120% to < 140% %BMIp95; class 3, ≥140% %BMIp95; classes 2 and 3 were considered severe obesity.^14^

### 2.1 Analyses

The frequency and percentage of patients overall, and for *normal BP* and *high BP* groups are presented. To evaluate associations with sex, age group, race/ethnicity, insurance group, age, %BMIp95, and HtZ, as appropriate, Pearson's chi‐square test was used to compare binary or nominal categorical variables, and the Kruskal‐Wallis test was used to compare continuous variables or ordinal categorical variables across BP group status. Additionally, for each BP interpretation category (normal, elevated, stage 1, stage 2) descriptive data for these same categorical and continuous variables are presented.

A mixed‐effects logistic regression model, with site as a random effect to account for multi‐site sampling, was used to evaluate the associations of factors with BP group status (*normal BP* vs. *high BP* group). Factors in the model included categorical variables of sex, age group, race/ethnicity, insurance group, and obesity class group. To further focus on youth with BP interpreted as stage 1 or stage 2 hypertension, a second mixed‐effects logistic regression model applied the same methods to examine associations of factors with BP interpretation group status between the combined normal BP and elevated BP groups (*normal/elevated* group) versus the group of youth with stage 1 or stage 2 hypertension (*stage 1/stage 2* group). Patients with missing race/ethnicity or insurance group were excluded from logistic regression analyses.

Frequency of BP interpretation groups by site are reported. All analyses were performed using SAS software version 9.4 (SAS Institute Inc., Cary, NC, USA).

## 3 RESULTS

Data on 7943 patients were analyzed; 54% were females. Patients were most often either Hispanic (32%) or White non‐Hispanic (NH) (39%) and 59% had public health insurance (Table 1). Mean age was 11.7 years (SD 3.3); about half (47%) of patients were aged 12–18 years; a majority (73%) had severe obesity. There were 35 sites contributing data with a median of 131 patients per site in analyses (interquartile range [IQR], 56–223 patients; range, 2–1239 patients).

**TABLE 1:** Patient characteristics by blood pressure status group

				Grouped by BP Status				
		Overall (*N* = 7943)		*Normal BP* (*N* = 4061, 51.1%)		*High BP* ^a^ (N = 3882, 48.9%)		
Variable	Level	*n*	%	*n*	%	*n*	%	*p*‐value^c^
**Categorical variables**								
Age group, years^d^	3–5	367	4.6	192	52.3	175	47.7	<.0001
	6–10	1296	16.3	711	54.9	585	45.1	
	9–11	2547	32.1	1380	54.2	1167	45.8	
	12–14	2197	27.7	1153	52.5	1044	47.5	
	15–17	1536	19.3	625	40.7	911	59.3	
Sex	Male	3668	46.2	1704	46.5	1964	53.5	<.0001
	Female	4275	53.8	2357	55.1	1918	44.9	
Race/ethnicity	Black NH	1495	18.8	810	54.2	685	45.8	.0001
	Hispanic	2503	31.5	1304	52.1	1199	47.9	
	White NH	3129	39.4	1557	49.8	1572	50.2	
	Other and multiracial NH	429	5.40	228	53.1	201	46.9	
	Unknown	387	4.9	162	41.9	225	58.1	
Primary health insurance	Private	2331	29.3	1155	49.6	1176	50.4	.0127
	Public	4669	58.8	2381	51.0	2288	49.0	
	Self‐pay/none	65	0.8	33	50.8	32	49.2	
	Unknown	878	11.1	492	56.0	386	44.0	
Obesity Class^b^	Class 1	2166	27.3	1350	62.3	816	37.7	<.0001
	Class 2	2854	35.9	1543	54.1	1311	45.9	
	Class 3	2923	36.8	1168	40.0	1755	60.0	
**Continuous variables** ^d^								
Age, years	Median	7943	11.7	4061	11.5	3882	12.0	<.0001
	25th		9.5		9.3		9.6	
	75th		14.3		13.8		14.8	
	Mean		11.7		11.5		12.0	
	STD		3.3		3.2		3.4	
	Min		3.0		3.0		3.0	
	Max		18.0		18.0		18.0	
%BMIp95	Median	7943	132.1	4061	128.3	3882	136.9	<.0001
	25th		118.8		115.9		122.3	
	75th		148.5		143.0		154.8	
	Mean		136.6		131.8		141.6	
	STD		25.4		22.5		27.2	
	Min		100.0		100.0		100.0	
	Max		505.2		505.2		376.8	
Height‐for‐age z‐score	Median	7943	0.8	4061	0.8	3882	0.8	.4454
	25th		0.1		0.1		0.1	
	75th		1.5		1.5		1.5	
	Mean		0.8		0.8		0.8	
	STD		1.1		1.1		1.1	
	Min		‐3.7		‐3.7		‐3.6	
	Max		3.7		3.7		3.7	

All tests treat the column variable as nominal.

*Abbreviations*: BP, blood pressure; NH, non‐Hispanic; BMI, body mass index; %BMIp95, percentage of the 95^th^ BMI percentile.

^a^ *High BP* includes elevated, stage 1 and stage 2.

^b^ Obesity Class: class 1, 100% to < 120% %BMIp95; class 2, 120% to < 140% %BMIp95; class 3, ≥140% %BMIp95.

^c^ *P*‐values are based on Pearson chi‐square tests for all categorical row variables.

^d^ *P*‐values for comparing ordinal categorical variables and continuous variables are based on chi‐square rank based group means score statistics. This is equivalent to Kruskal‐Wallis tests. All tests treat the column variable as nominal.

Nearly half of patients (3882/7943, 48.9%) had BP interpreted as *high BP* (18.9% elevated, 23.9% stage 1, and 6.0% stage 2) and 51.1% had *normal BP*. *High BP* was found for 60.0% of youth with class 3 obesity, 45.9% with class 2 obesity, and 37.7% with class 1 obesity, (Table 1). Highest frequency of *high BP* status occurred in older teens, males, and for White NH youth and those of unknown race/ethnicity status (Table 1). Descriptive data for continuous variables (age, %BMIp95, and HtZ) are also presented in Table 1. Table S1 (online) presents these data for four BP interpretation groups: normal BP (51.1%); elevated BP (18.9%); stage 1 hypertension (23.9%); and stage 2 hypertension (6.0%).

As a next step, the first multivariate analysis evaluated the 6752 patients with complete data; 1191 patients (15.0%) were excluded due to missing race/ethnicity and/or missing insurance group. The first analysis evaluated factors associated with being in the *high BP* (*n* = 3311, 49.0%) versus the *normal BP* group (*n* = 3441, 51.0%). Youth with severe obesity and males were more likely to have *high BP* (Table 2). Younger age groups (3–5, 6–8, 9–11, 12–14 years) were less likely to have *high BP* compared to youth ages 15–17 years. White NH youth were more likely to have *high BP* status as compared to Black NH youth, but were similar to Hispanic and Other/Multiracial NH groups. Insurance group did not significantly predict BP group status.

**TABLE 2:** Generalized linear multivariable model examining association of characteristics with being in the high BP group compared to normal BP group based on 2017 AAP CPG BP interpretations^7^

	Univariate analysis**^+^**		Multivariate analysis**^†^**	
Characteristics	OR (95% CI)	*p* value	OR (95% CI)	*p* value
**Sex**				
Male	1.44 (1.30, 1.59)	<.0001	1.41 (1.27, 1.56)	<.0001
Female	Ref		Ref	
**Age groups**				
3–5 years	0.69 (0.54, 0.89)	.0042	0.65 (0.50, 0.85)	.0012
6–8 years	0.57 (0.48, 0.68)	<.0001	0.57 (0.48, 0.68)	<.0001
9–11 years	0.60 (0.52, 0.69)	<.0001	0.63 (0.54, 0.73)	<.0001
12–14 years	0.64 (0.55, 0.74)	<.0001	0.64 (0.55, 0.75)	<.0001
15–17 years	Ref		Ref	
**Race/ethnicity**				
Black NH	0.89 (0.77, 1.02)	.0972	0.84 (0.72, 0.97)	.0212
Hispanic	0.86 (0.75, 0.98)	.0253	0.91 (0.79, 1.05)	.1908
Other and multiracial NH	0.92 (0.73, 1.15)	.4688	0.97 (0.77, 1.22)	.7735
White NH	Ref		Ref	
**Insurance type**				
Public	0.98 (0.88, 1.09)	.6829	0.95 (0.85, 1.07)	.4203
Self‐pay/none	0.98 (0.58, 1.65)	.9463	1.04 (0.61, 1.78)	.8767
Private	Ref		Ref	
**Obesity class** ^**a**^				
Class 3	2.46 (2.16, 2.80)	<.0001	2.39 (2.10, 2.73)	<.0001
Class 2	1.43 (1.26, 1.62)	<.0001	1.42 (1.25, 1.62)	<.0001
Class 1	Ref		Ref	

*Abbreviations*: BP, blood pressure; AAP CPG, American Academy of Pediatrics Clinical Practice Guideline^7^; OR, odds ratio; Ref, reference; NH, non‐Hispanic.

^a^ Obesity Class: class 1, 100% to < 120% %BMIp95; class 2, 120% to < 140% %BMIp95; class 3, ≥140% %BMIp95.

^+^ Includes site as a random effect.

^†^ Includes site as a random effect and other characteristics in the model.

The next multivariate analyses again evaluated the 6752 patients with complete data to examine factors associated with being in the *stage 1/stage 2 BP* group (*n* = 2021, 29.9%) as compared to the *normal/elevated BP* group (*n* = 4731, 70.1%). Youth with severe obesity and males were more likely to be in the *stage 1/stage 2 BP* group (Table 3). However, as compared to youth ages 15–17 years, only those in the 12‐14‐year age group were less likely to be in the *stage 1/stage 2 BP* group; with no significant differences found between other age groups compared to youth ages 15–17 years. Neither race/ethnicity nor insurance group significantly predicted BP group status.

**TABLE 3:** Generalized linear multivariable model examining association of characteristics with being in the Stage 1/Stage 2 BP group compared to the normal/elevated BP group based on 2017 AAP CPG BP interpretations^7^

	Univariate analysis**^+^**		Multivariate analysis**^†^**	
Characteristics	OR (95% CI)	*p* value	OR (95% CI)	*p* value
**Sex**				
Male	1.31 (1.18, 1.46)	<.0001	1.25 (1.12, 1.40)	<.0001
Female	Ref		Ref	
**Age groups**				
3–5 years	1.07 (0.81, 1.39)	.6443	1.01 (0.77, 1.33)	.9501
6–8 years	0.96 (0.80, 1.15)	.6503	0.98 (0.81, 1.17)	.7943
9–11 years	0.89 (0.76, 1.04)	.1486	0.97 (0.82, 1.13)	.6804
12–14 years	0.68 (0.58, 0.79)	<.0001	0.69 (0.58, 0.81)	<.0001
15–17 years	Ref		Ref	
**Race/ethnicity**				
Black NH	0.95 (0.82, 1.11)	.5515	0.87 (0.74, 1.02)	.0855
Hispanic	0.83 (0.73, 0.95)	.0049	0.91 (0.77, 1.06)	.2228
Other and multiracial NH	1.02 (0.79, 1.30)	.8976	1.05 (0.81, 1.35)	.7298
White NH	Ref		Ref	
**Insurance type**				
Public	1.05 (0.93, 1.18)	.4558	0.99 (0.88, 1.13)	.9205
Self‐pay/none	0.91 (0.51, 1.62)	.7404	0.95 (0.52, 1.71)	.8531
Private	Ref		Ref	
**Obesity class** ^*^				
Class 3	2.77 (2.39, 3.21)	<.0001	2.75 (2.37, 3.20)	<.0001
Class 2	1.54 (1.32, 1.79)	<.0001	1.54 (1.32, 1.79)	<.0001
Class 1	Ref		Ref	

*Abbreviations*: BP, blood pressure; AAP CPG, American Academy of Pediatrics Clinical Practice Guideline^7^; OR, odds ratio; Ref, reference; NH, non‐Hispanic.

* Obesity Class: class 1, 100% to < 120% %BMIp95; class 2, 120% to < 140% %BMIp95; class 3, ≥140% %BMIp95.

^+^ Includes site as a random effect.

^†^ Includes site as a random effect and other characteristics in the model.

The percentage of patients with *high BP* varied between sites. For sites with > 100 patients in the analyses (*n* = 21), the median percentage of patients with *high BP* at the initial visit was 50.4% (IQR 36.4–56.1%; range 25.5–80.7%). For sites with ≤100 patients in the analyses (*n* = 14), the median percentage of patients with *high BP* at the initial visit was 63.5% (IQR 54.9–76.6%; range 30.6–100%).

## 4 DISCUSSION

Hypertension is a serious comorbidity associated with obesity across all ages, including children.^15^, ^16^ This study found that nearly half of treatment‐seeking youth with obesity had *high BP*. This rate is higher than previous studies. For example, Reinehr and coworkers, reported that among a sample of treatment‐seeking youth aged 4–18 years with overweight and obesity and having BP assessed using a standardized protocol, 37% had BP > 95^th^ percentile compared to a large US reference sample.^17^ A previous report found that for youth 12–17 years in POWER, systolic and diastolic BP values were significantly higher as compared to a sample of youth with obesity selected from national survey evaluations.^18^ Differing rates of severe obesity between studies may be the reason for variation in the rates of *high BP*. In the Reinehr and coworkers study, 45% of patients had severe obesity (defined for the study as BMI > 99.5^th^ percentile)^17^ and in national surveys approximately one‐third of patients had severe obesity.^5^, ^8^, ^18^ In this report and prior POWER reports severe obesity status was reported for three‐quarters of the patients^11^, ^18^; among youth with most severe (class 3) obesity ∼60% had *high BP*. Youth with hypertension and obesity are often referred for PWM services due to the need for a multi‐disciplinary team approach available in these centers,^19^, ^20^ and that may also have been a contributing factor to the high rates of *high BP* observed in this sample.

Many of the characteristics significantly associated with *high BP* in the initial visit for treatment‐seeking youth with obesity were as expected. Youth with severe obesity status were at increased risk for *high BP*. Reinehr and coworkers found that obesity severity is an important risk for *high BP*.^17^ Jackson and coworkers, using 2013–2016 NHANES data, reported *high BP* for 27.5% of youth with obesity, with highest frequency among youth with severe obesity (30.6%).^8^ Older youth were significantly more likely to have *high BP*. Similar results were reported by Reinehr and coworkers.^17^ Several large community samples^21^ ^,^ ^22^ and a recent report of national data^9^ have also reported increased risk for *high BP* as children age.

Males more frequently had *high BP* as compared to females, as was also found in national surveys.^9^, ^23^ The AAP CPG accounts for expected sex‐related BP differences for interpretations for youth ages 3–12 years, but both male and female youth ages 13–17 are evaluated using the same cut points.^7^ This may have also been a contributing factor to our findings, since about 45% of youth in this evaluation were ages 12–17 years.

This study did not examine characteristics associated with the *high BP* group for males and females separately but found that when males and females were considered as a group, Black NH youth had lower risk for *high BP* as compared to White NH youth. Other studies have reported disparate findings on racial/ethnic differences in likelihood of *high BP*. A study of BP elevation in youth with obesity found higher risk for Hispanic males as compared to White males, with similar risk for Black and White males and no racial/ethnic differences in risk for BP elevation among females.^24^ A large community sample also reported slightly lower risk for elevated BP among African American youth as compared to White youth.^25^ In contrast, an evaluation of a national sample of youth ages 8–12 years found higher prevalence of *high BP* among Asian NH youth, as compared to White NH youth with no significant difference by race/ethnicity for youth ages 13–17 years.^9^ It has been suggested that racial/ethnic differences are developed during adolescence,^7^, ^26^ so expected difference would depend on ages of the youth being studied.

There was no difference in the frequency of *high* BP between those with public and private insurance. In contrast, a community sample reported slightly higher odds for elevated BP among those with public insurance or uninsured, as compared to commercially insured patients.^25^

The second logistic regression examined factors associated with having stage 1 or stage 2 hypertension as compared to patients in the *normal/elevated BP* group. Compared to the first logistic regression, findings were similar for risks of severe obesity and being male, but findings for age group and race/ethnicity differed. Only youth ages 12–14 years were less likely to have stage 1 or stage 2 hypertension compared to youth ages 15–17 years; all other age groups were similar to youth ages 15–17 years. This indicates that in the first logistic regression youth ages 2–11 years with BP assessed as elevated were driving the difference found for the comparison with the 15–17‐year age group. Since BP increases with age,^26^ youth ages 13–14 years evaluated by the same standard at youth 15–17 years would be expected to have lower likelihood of being in the *stage 1/stage 2 BP* group, and this may have contributed to the findings reported. Youth ages 3–12 years have BP interpretations evaluated in relationship to age and height and that strategy provides gradual change in BP interpretation cut point that accounts for the gradual rise in BP as youth age. Also, in this second logistic regression, having fewer patients, neither race/ethnicity nor insurance were significant factors predicting being in the *stage 1/stage 2 BP* group.

*High BP* may prompt the need for further evaluation, such as repeated BP assessments at future visits, ambulatory blood pressure monitoring, echocardiogram evaluations, or require prompt initiation of antihypertensive medications.^7^ PWM clinicians should incorporate initial recommended hypertension management strategies such as nutritional and activity counseling as core components of an intensive lifestyle intervention.^7^ An effective nutritional strategy to reduce incidence of hypertension in youth has been application of a Dietary Approaches to Stop Hypertension (DASH)‐style diet.^27^ This diet includes: increased intake of fruits, vegetables, nuts, legumes and whole grains; low‐fat dairy products; lean meats, poultry, and fish (while limiting red and processed meats); and limiting sweets, sugar‐containing beverages and sodium.^27^ Medical interventions in PWM can lead to improvements in BP,^28^, ^29^ even over periods as short as 6 months.^29^ In adults, a weight loss of 1 kg was found to be associated with ‐1.05 mm Hg and ‐0.92 mm Hg changes in systolic and diastolic BP, respectively.^30^ Among adolescents with overt hypertension there are also several factors (eg, insulin resistance, levels of pro‐inflammatory cytokines) that indicate higher risk of concurrent subclinical disease, persistent adult hypertension, and adult cardiovascular disease.^31^ Clinical PWM programs may benefit substantially from more aggressive antihypertensive pharmacological treatments and referral to other subspecialists (eg, nephrologist or cardiologist) for further evaluation and pharmacotherapy.

These data were obtained during clinical care and show substantial between‐site variability in frequency of youth with *high BP*. The between‐site variability may reflect, to some degree, differences in patient populations across sites, but also highlights the importance of ensuring use of recommended protocols for BP measurement,^7^ including adequate BP cuff size and repeated manual measurements, if necessary. A study of outpatient visits at one pediatric tertiary care institution reported 36% of BP readings to be high, but across institutional subspecialties this varied from 12.4% to 65.2%, with the PWM program having *high* BP frequency at rates similar to the Kidney Disease division (∼48%).^32^

BP data for this study were collected during clinical care and entered the POWER database as a single BP measurement; therefore, the frequency of sites performing single versus duplicate BP measurements is unknown. A single BP measurement value does not fulfill guideline requirements for repeated measurements at visits.^7^ When an initial BP value is interpreted as elevated, two additional auscultatory measurements should be taken and averaged to define the BP category.^7^ Repeated BP measurements may not be a routine part of primary care visits,^33^ and repeated measurements may be particularly important for youth with overweight or obesity.^34^, ^35^ Repeated measurements better predict longer‐term BP status.^36^ Additionally, to assign a diagnosis of hypertension requires identification of elevated BP measurements at three different occasions.^7^, ^15^

There is a substantial selection bias for youth in this evaluation that can explain differences with data from random samples from a general population. Treatment‐seeking youth more frequently have severe obesity, a risk for hypertension, compared to general population samples. Additionally, treatment‐seeking youth with obesity may be at substantially higher risk for hypertension, as BP measurements at a primary care visit may have been the factor initiating a PWM program referral. Further study will be needed to identify which youth will fulfill hypertension criteria across repeated measurement encounters. For some youth, additional evaluations may be needed to better understand BP in clinical and home settings. Ambulatory BP monitoring may be needed to identify white‐coat hypertension, possibly induced by the stress of a clinical encounter to address weight issues, as a contributing factor to BP value elevation.^37^ Maggio conducted casual and ambulatory BP evaluations on 44 youth with obesity (mean age 8.9 years, mean BMI 25.3 kg/m^2^), finding systolic ambulatory hypertension in 46.7%, with 55% of those having normal casual BP measurements.^38^

### 4.1 Strengths and limitations

These data present information on nearly 8000 youth presenting for PWM care with the majority having severe obesity. Such information can foster the development of multidisciplinary strategies to address obesity and hypertension and help PWM programs to realize the high frequency at which they will need to optimally measure BP and treat elevated BP with appropriate lifestyle changes and pharmacotherapy.^31^

While a limitation within POWER is the lack of information about BP measurement validation, these data were obtained during clinical care and highlight the diversity of the frequency of *high BP* between PWM programs. Individual PWM programs may wish to examine the frequency of *high BP* for their program in comparison to these data and, as necessary, review adherence to protocols for BP measurement.

Some patients may have already had a diagnosis of hypertension and were receiving antihypertensive medications for treatment at the time of their initial PWM visit. Diagnoses and medications were not uniformly collected and thus were not analyzed. Clearly, given this high frequency of *high BP* in this study, many PWM patients may need additional support to address hypertension and PWM clinicians will need to monitor if youth are taking antihypertensive medications as prescribed. Despite limitations, these data identify the high frequency of and important considerations for risk of *high BP* among treatment‐seeking youth with obesity which can prompt PWM programs to develop strategies to address *high BP* in the youth they serve.

## 5 CONCLUSIONS

Nearly half of youth seeking PWM obesity care had BP values classified as *high*. Males, older teens, and youth with severe obesity were at highest risk for *high BP*. There were substantial differences in prevalence of *high BP* between POWER sites. Standardized, protocol‐driven assessments and management of *high BP* should be key areas of focus for PWM programs.

## AUTHOR CONTRIBUTIONS

All authors participated in study conception and design. Eileen King provided data cleaning. Asheley Skinner and Haolin Xu conducted analyses. All authors participated in interpretation of data. Helen Binns drafted the manuscript. Helen Binns, Madeline Joseph, Sarah Hampl, and Shelley Kirk conducted literature searches. All authors provided critical revisions and approved the final version.

## CONFLICTS OF INTEREST

All authors have declared that they have no conflicts of interest.

## Supporting information

SUPPORTING INFORMATION
