# Supplementary material for: Elevated blood pressure in youth in pediatric weight management programs in the Pediatric Obesity Weight Evaluation Registry (POWER)
Source: J Clin Hypertens (Greenwich). 2022 Jan 31;24(2):122–30. doi: 10.1111/jch.14423 (PMC8845448; doi:10.1111/jch.14423)
Supplement: Supplementary file 1 — SUPPORTING INFORMATION [file JCH-24-122-s001.docx]

**Supplemental Table 1**: Subject Characteristics by Blood Pressure Interpretation Group

|  |  | **Overall**  **(N=7943)** | | **2017 AAP CPG Interpretation Group**^7^ | | | | | | | |
| --- | --- | --- | --- | --- | --- | --- | --- | --- | --- | --- | --- |
| **Variable** | **Level** |  |  | **Normal BP**  **(N=4061, 51.1%)** | | **Elevated BP**  **(N=1505, 18.9%)** | | **Stage 1 HTN**  **(N=1899, 23.9%)** | | **Stage 2 HTN**  **(N=478, 6.0%)** | |
|  |  | **n** | **%** | **n** | **%** | **n** | **%** | **n** | **%** | **n** | **%** |
| **Categorical Variables** | |  |  |  |  |  |  |  |  |  |  |
|  |  |  |  |  |  |  |  |  |  |  |  |
| Age group, years | 3 -5 | 367 | 4.6 | 192 | 52.3 | 57 | 15.5 | 102 | 27.8 | 16 | 4.4 |
|  | 6 -10 | 1296 | 16.3 | 711 | 54.9 | 179 | 13.8 | 344 | 26.5 | 62 | 4.8 |
|  | 9 -11 | 2547 | 32.1 | 1380 | 54.2 | 397 | 15.6 | 665 | 26.1 | 105 | 4.1 |
|  | 12 -14 | 2197 | 27.7 | 1153 | 52.5 | 489 | 22.3 | 429 | 19.5 | 126 | 5.7 |
|  | 15 -17 | 1536 | 19.3 | 625 | 40.7 | 383 | 24.9 | 359 | 23.4 | 169 | 11.0 |
|  |  |  |  |  |  |  |  |  |  |  |  |
| Sex | Male | 3668 | 46.2 | 1704 | 46.5 | 750 | 20. 5 | 941 | 25.7 | 273 | 7.4 |
|  | Female | 4275 | 53.8 | 2357 | 55.1 | 755 | 17.7 | 958 | 22.4 | 205 | 4.8 |
|  |  |  |  |  |  |  |  |  |  |  |  |
| Race/ Ethnicity | Black NH | 1495 | 18.8 | 810 | 54.2 | 261 | 17.5 | 352 | 23.6 | 72 | 4.8 |
|  | Hispanic | 2503 | 31.5 | 1304 | 52.1 | 477 | 19.1 | 573 | 22.9 | 149 | 6.0 |
|  | White NH | 3129 | 39.4 | 1557 | 49.8 | 621 | 19.9 | 749 | 23.9 | 202 | 6.5 |
|  | Other and Multiracial NH | 429 | 5.4 | 228 | 53.2 | 71 | 16.6 | 111 | 25.9 | 19 | 4.4 |
|  | Unknown | 387 | 4.9 | 162 | 41.9 | 75 | 19.4 | 114 | 29.5 | 36 | 9.3 |
|  |  |  |  |  |  |  |  |  |  |  |  |
| Primary Health Insurance | Private | 2331 | 29.4 | 1155 | 49.7 | 459 | 19.7 | 574 | 24.6 | 143 | 6.1 |
|  | Public | 4669 | 58.8 | 2381 | 51.0 | 879 | 18.8 | 1123 | 24.1 | 286 | 6.1 |
|  | Self-Pay/None | 65 | 0.8 | 33 | 50.8 | 14 | 21.5 | 13 | 20.0 | 5 | 7.7 |
|  | Unknown | 878 | 11.1 | 492 | 56.0 | 153 | 17.4 | 189 | 21.5 | 44 | 5.0 |
|  |  |  |  |  |  |  |  |  |  |  |  |
| Obesity Class^∇^ | Class 1 | 2166 | 27.3 | 1350 | 62.3 | 393 | 18.1 | 375 | 17.3 | 48 | 2.2 |
|  | Class 2 | 2854 | 35.9 | 1543 | 54.1 | 538 | 18.8 | 639 | 22.4 | 134 | 4.7 |
|  | Class 3 | 2923 | 36.8 | 1168 | 40.0 | 574 | 19.6 | 885 | 30.3 | 296 | 10.1 |
|  |  |  |  |  |  |  |  |  |  |  |  |
| **Continuous Variables** | |  |  |  |  |  |  |  |  |  |  |
|  |  |  |  |  |  |  |  |  |  |  |  |
| Age, years | Median | 7943 | 11.7 | 4061 | 11.5 | 1505 | 12.7 | 1899 | 11.3 | 478 | 13.3 |
|  | 25th |  | 9.5 |  | 9.3 |  | 10.3 |  | 9.1 |  | 10.3 |
|  | 75th |  | 14.3 |  | 13.8 |  | 15.0 |  | 14.0 |  | 15.9 |
|  | Mean |  | 11.7 |  | 11.5 |  | 12.4 |  | 11. 5 |  | 12.9 |
|  | STD |  | 3.3 |  | 3.2 |  | 3.3 |  | 3.3 |  | 3.5 |
|  | Min |  | 3.0 |  | 3.0 |  | 3.0 |  | 3.0 |  | 3.2 |
|  | Max |  | 18.0 |  | 18.0 |  | 18.0 |  | 18.0 |  | 18.0 |
|  |  |  |  |  |  |  |  |  |  |  |  |
| %BMIp95 | Median | 7943 | 132.1 | 4061 | 128.3 | 1505 | 133.4 | 1899 | 137.5 | 478 | 147.8 |
|  | 25th |  | 118.8 |  | 115.9 |  | 119.4 |  | 123.4 |  | 129.9 |
|  | 75th |  | 148.5 |  | 143.0 |  | 148.8 |  | 155.3 |  | 171.2 |
|  | Mean |  | 136.6 |  | 131.8 |  | 136.7 |  | 142.3 |  | 154.0 |
|  | STD |  | 25.4 |  | 22.5 |  | 24.1 |  | 26.7 |  | 33.5 |
|  | Min |  | 100.0 |  | 100.0 |  | 100.0 |  | 100.4 |  | 100.9 |
|  | Max |  | 505.2 |  | 505.2 |  | 300.0 |  | 357.3 |  | 376.8 |
|  |  |  |  |  |  |  |  |  |  |  |  |
| Height-for-age z-score | Median | 7943 | 0.8 | 4061 | 0.8 | 1505 | 0.8 | 1899 | 0.9 | 478 | 0.8 |
|  | 25th |  | 0.1 |  | 0.1 |  | -0.1 |  | 0.2 |  | 0.0 |
|  | 75th |  | 1.5 |  | 1.5 |  | 1.5 |  | 1.6 |  | 1.6 |
|  | Mean |  | 0.8 |  | 0.8 |  | 0.7 |  | 0.9 |  | 0.8 |
|  | STD |  | 1.1 |  | 1.1 |  | 1.1 |  | 1.1 |  | 1.2 |
|  | Min |  | -3.7 |  | -3.7 |  | -3.3 |  | -3.6 |  | -3.5 |
|  | Max |  | 3.7 |  | 3.7 |  | 3.6 |  | 3.7 |  | 3.5 |
|  |  |  |  |  |  |  |  |  |  |  |  |

AAP CPG, American Academy of Pediatrics Clinical Practice Guideline^7^; BP, blood pressure; HTN, hypertension; NH, non-Hispanic; BMI, body mass index; %BMIp95, percent of the 95^th^ BMI percentile

^∇^Obesity Class: Class 1 = 100% ≥%BMIp95<120%, Class 2 120% ≥%BMIp95<140%, Class 3 %BMIp95≥140%
